# Arm swing responsiveness to dopaminergic medication in Parkinson’s disease depends on task complexity

**DOI:** 10.1038/s41531-021-00235-1

**Published:** 2021-10-05

**Authors:** Elke Warmerdam, Robbin Romijnders, Clint Hansen, Morad Elshehabi, Milan Zimmermann, Florian G. Metzger, Anna-Katharina von Thaler, Daniela Berg, Gerhard Schmidt, Walter Maetzler

**Affiliations:** 1 Department of Neurology, Kiel University, Kiel, Germany; 2 Faculty of Engineering, Kiel University, Kiel, Germany; 3 Department of Neurodegeneration, Hertie Institute for Clinical Brain Research, University of Tübingen, Tübingen, Germany; 4 German Center for Neurodegenerative Diseases, Tübingen, Germany; 5 Department of Psychiatry and Psychotherapy, University Hospital of Tübingen, Tübingen, Germany; 6 Geriatric Center, University Hospital of Tübingen, Tübingen, Germany; 7 Vitos Hospital of Psychiatry and Psychotherapy Haina, Haina, Germany

**Keywords:** Parkinson's disease, Neurological manifestations, Parkinson's disease

## Abstract

The evidence of the responsiveness of dopaminergic medication on gait in patients with Parkinson’s disease is contradicting. This could be due to differences in complexity of the context gait was in performed. This study analysed the effect of dopaminergic medication on arm swing, an important movement during walking, in different contexts. Forty-five patients with Parkinson’s disease were measured when walking at preferred speed, fast speed, and dual-tasking conditions in both OFF and ON medication states. At preferred, and even more at fast speed, arm swing improved with medication. However, during dual-tasking, there were only small or even negative effects of medication on arm swing. Assuming that dual-task walking most closely reflects real-life situations, the results suggest that the effect of dopaminergic medication on mobility-relevant movements, such as arm swing, might be small in everyday conditions. This should motivate further studies to look at medication effects on mobility in Parkinson’s disease, as it could have highly relevant implications for Parkinson’s disease treatment and counselling.

## Introduction

Dopaminergic medication is the most common treatment for people with Parkinson’s disease. It is highly effective in improving Parkinson’s disease-related symptoms such as bradykinesia, rigidity, and tremor, as has been shown, for example, with the unified Parkinson’s disease rating scale (UPDRS), its revised version (MDS-UPDRS), and other established clinical scales^1–4^.

However, contradicting results were found concerning the effect of dopaminergic medication on gait deficits associated with Parkinson’s disease. Only gait speed, stride length, and stride velocity have consistently shown an increase with medication in multiple studies with different disease severities^2,5,6^, study protocols^2,7–10^, and measurement equipments^2,5,8,10–13^. The effects of dopaminergic medication on other gait parameters are not entirely clear. For example, although one relatively large study found an increased cadence (steps per minute) with dopaminergic medication^5^, four others—with comparable cohort characteristics—did not^7,8,10,13^. One study found a decrease instance time^11^ but another study—again with comparable cohort characteristics—did not^5^. Contradicting results were also found for double limb support (for example, one study found no significant change^5^, where other studies found a decrease with medication^10,11,14^) and gait variability (three studies found no significant change^8,11,15^, four studies found a decrease with medication^8,9,13,15^). Similarly, there are contradicting results concerning the effect of dopaminergic medication on arm swing parameters in Parkinson’s disease. For example, arm swing asymmetry only decreased with medication in one^16^ but not in another study^5^.

Brain activity differs with the complexity of walking tasks and with neurological pathologies^17^. We therefore hypothesize that at least some of the above-mentioned contradicting results may be explained by differences in the context where the respective walking task is performed. This hypothesis is, at least indirectly, supported by studies that found an effect of task complexity on the effect of medication on certain gait parameters (e.g., gait speed^18^ and stride time variability^19^). Moreover, two studies reported a change in the difference of walking parameters between Parkinson’s disease and controls, depending on the walking paradigm (between preferred and fast walking condition: gait speed, swing velocity, step time, and swing time; between preferred and dual-task condition: stride length and percentage swing time^20,21^). These differences in the response of mobility patterns to different stimuli and demands could have highly relevant implications for Parkinson’s disease treatment and counselling, as human behavior depends on the use of highly diverse mobility strategies^22^.

We therefore measured in this study the effect of dopaminergic medication on a specific movement, i.e., arm swing, during preferred, fast and dual-task walking. We then compared the delta of medication ON minus OFF, of different arm swing parameters between the different walking conditions. We chose arm swing because arm swing (i) is relatively easy and very reliable to measure^23^, (ii) is influenced by cognitive dual-tasks^24–26^ that occur regularly in daily life, (iii) is influenced by Parkinson’s disease (smaller arm swing amplitudes and more asymmetry compared to controls)^27–29^, and (iv) is influenced by dopaminergic medication. For example, arm swing amplitude and angular velocity increase with medication^5,16,30^.

## Results

### Changes in arm swing with dopaminergic medication

Demographics and task performance of the included Parkinson’s disease patients are provided in Tables 1 and 2.

**Table 1:** Demographics and disease characteristics (mean ± standard deviation (range)) of the participants.

Characteristics	Participants with Parkinson’s disease
n (male)	45 (30)
age [years]	65 ± 9 (46–84)
height [m]	1.73 ± 0.11 (1.55–1.93)
weight [kg]	77 ± 13 (53–107)
MoCA (0–30)	27 ± 2 (20–30)
Hoehn & Yahr (1–5)	2.0 ± 0.5 (HY1 = 5, HY1.5 = 2, HY2 = 32, HY2.5 = 2, HY3 = 4)
Disease duration [years]	5 ± 3 (1–10)
Levodopa equivalent dose [mg]	523 ± 379 (155–1630)
MDS-UPDRS III (0–132)	21 ± 9 (6–61)

*MDS-UPDRS III* motor part of the Movement Disorders Society-sponsored revision of the unified Parkinson’s disease rating scale.

**Table 2:** Performance (mean ± standard deviation (range)) of the participants.

Parameters	OFF medication	ON medication
Preferred gait speed [m/s]	1.34 ± 0.22 (0.88–1.87)	1.39 ± 0.19 (0.95–1.78)
Fast gait speed [m/s]	1.68 ± 0.25 (1.23–2.17)	1.75 ± 0.26 (1.26–2.52)
Dual-task gait speed [m/s]	1.29 ± 0.27 (0.68–1.80)	1.34 ± 0.27 (0.73–2.13)
Number of arm swings in preferred condition	87 ± 17 (51–128)	88 ± 18 (36–138)
Number of arm swings in fast condition	86 ± 17 (36–122)	86 ± 17 (51–130)
Number of arm swings in dual-task condition	89 ± 31 (35–231)	81 ± 16 (43–116)
Subtractions in single-task condition [*n*/min]	21 ± 13 (6–60)	23 ± 13 (4–46)
Subtractions in dual-task condition [*n*/min]	24 ± 12 (4–50)	22 ± 11 (9–61)
Subtraction mistakes in single-task condition [*n*/min]	1 ± 2 (0–9)	1 ± 2 (0–5)
Subtraction mistakes in dual-task condition [*n*/min]	2 ± 2 (0–8)	1 ± 3 (0–9)

*MoCA* Montreal cognitive assessment.

**Fig. 1:**
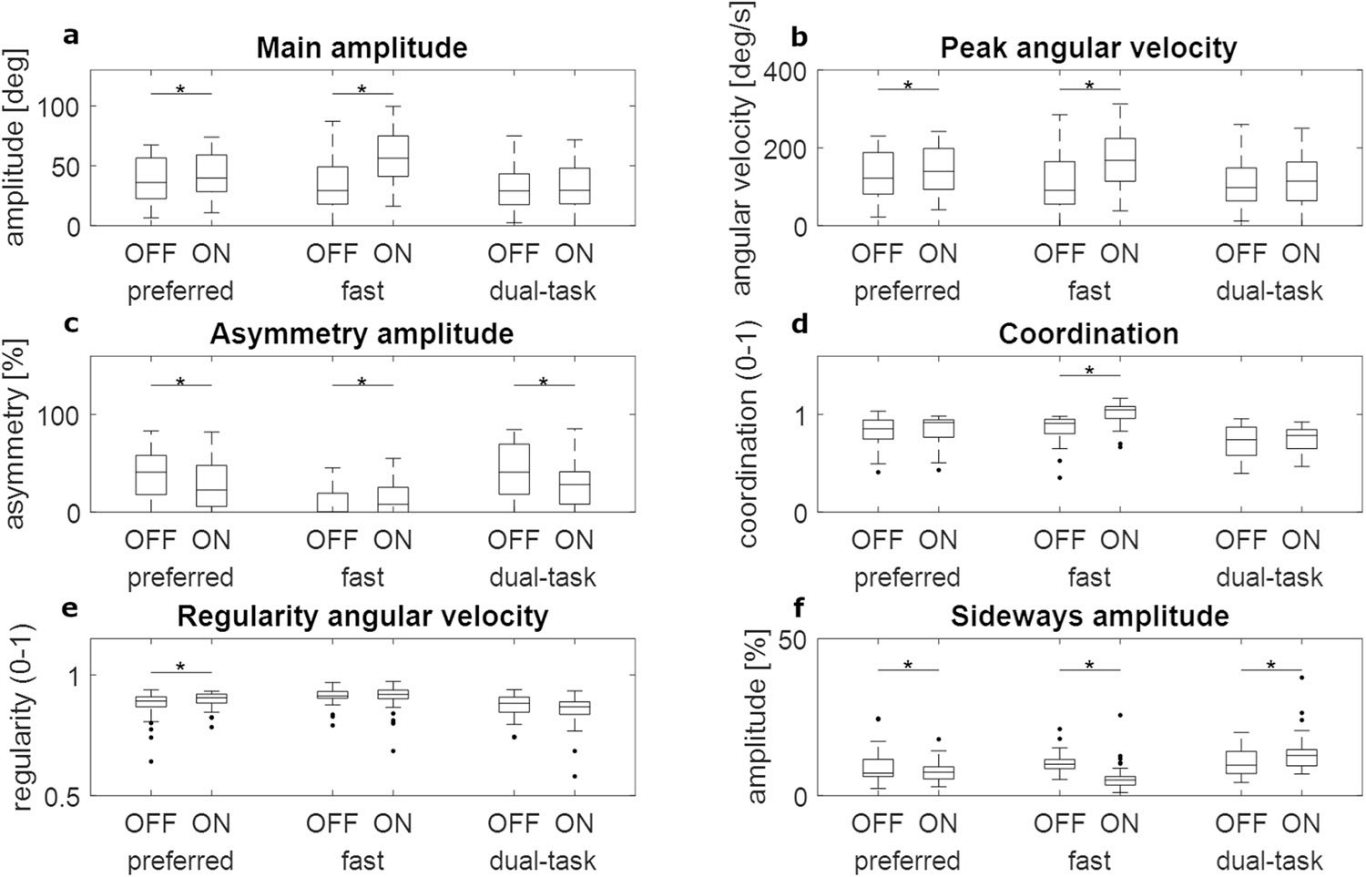
Arm swing parameters during the different medication states and different walking conditions. **a** Main amplitude, **b** Peak angular velocity, **c** Asymmetry of the amplitude, **d** Coordination between the arms, **e** Regularity of the angular velocity and **f** amplitude of the sideways arm swing. * above horizontal lines, connecting different box plots = *P* < 0.05 between medication states. All data are corrected for gait speed. Center line: median; box limits: upper and lower quartiles; whiskers: 1.5 × interquartile range.

The following changes of arm swing parameters due to dopaminergic medication were significant (see also Fig. 1 and Supplementary Table 1). Main amplitude and peak angular velocity increased with medication in the preferred and fast walking condition, but not in the dual-task condition. Amplitude asymmetry decreased with medication in the preferred and dual-task conditions, but not at fast speed. Arm swing coordination only increased in the fast walking condition. Regularity improved with medication only in the preferred condition. The sideways amplitude decreased with medication during the preferred and fast walking condition, but *increased* during the dual-task condition.

### Cognitive performance

**Fig. 2:**
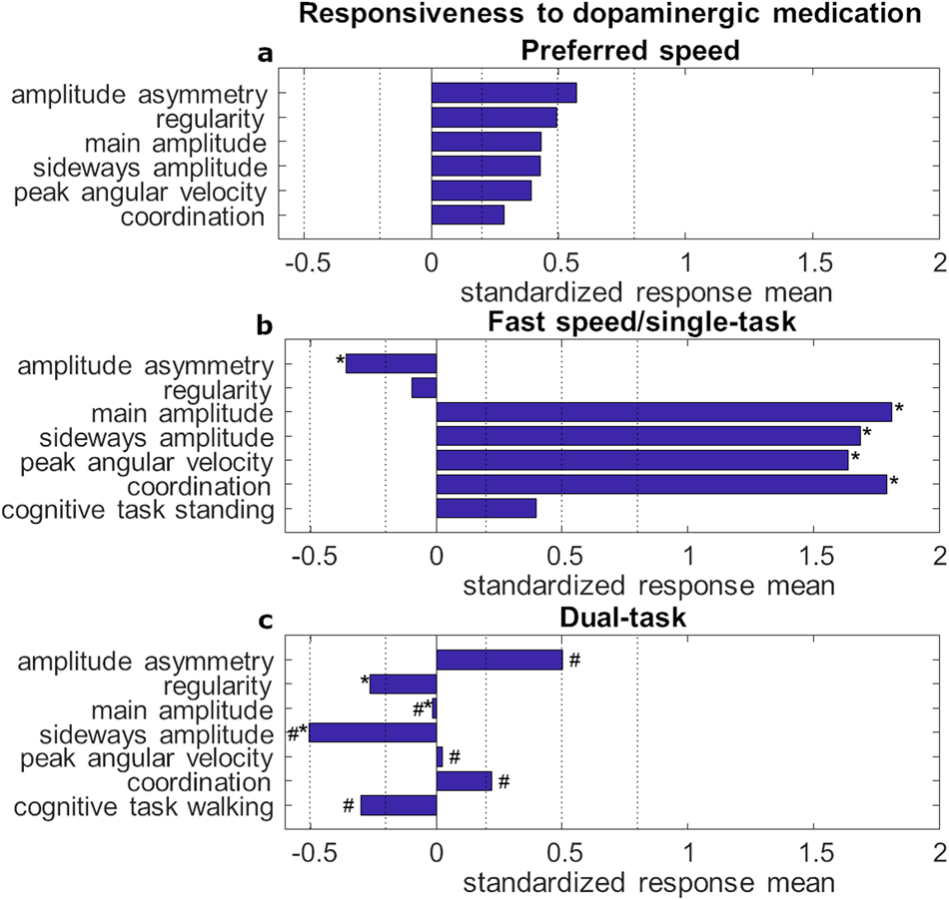
Responsiveness of the arm swing parameters and the cognitive subtraction task to dopaminergic medication. A positive standardized response mean (SRM) indicates an improvement with medication and a negative SRM a worsening with medication. **a** Walking at preferred speed, **b** Walking at fast speed and **c** Walking with dual-task. 0.20 ≤ SRM < 0.50 represents a small, 0.50 ≤ SRM < 0.80 a moderate and SRM ≥ 0.80 a large responsiveness to dopaminergic medication^5^. *significantly different from preferred speed; #significantly different from fast speed/single-task condition.

Cognitive performance as measured with subtractions per minute improved with medication during the single-task (*P* = 0.012), but not during the dual-task. The responsiveness to dopaminergic medication was significantly different between the single-task and dual-task (*P* = 0.005; Fig. 2). Moreover, cognitive dual-task costs were significantly different per medication state (*P* = 0.027), −29 % in OFF state and −12 % in ON state.

### Responsiveness of arm swing to dopaminergic medication

The degree of responsiveness of respective arm swing parameters to dopaminergic medication are shown in Fig. 2 for the 33 participants with a complete dataset. At preferred speed, the responsiveness to dopaminergic medication was moderate for amplitude asymmetry and small for all other arm swing parameters. At fast speed, the responsiveness to medication was large for main amplitude, peak angular velocity, coordination, and sideways amplitude (decrease), small for amplitude asymmetry, and negligible for regularity. The responsiveness to medication was small for the cognitive single-task. In the dual-task condition, the responsiveness to dopaminergic medication was moderate for amplitude asymmetry and sideways amplitude (increase), small for regularity, coordination, and cognitive performance, and negligible for main amplitude and peak angular velocity.

The following responses of arm swing parameters to dopaminergic medication were significantly different across the different walking conditions in Parkinson’s disease (special characters in Fig. 2 and Supplementary Table 2): In the fast walking condition, main amplitude, peak angular velocity, coordination, and sideways amplitude were significantly more responsive (i.e., better) and asymmetry was significantly less responsive (i.e., worse) than in the preferred walking condition. Regularity was not significantly different between these two conditions. In the dual-task walking condition, main amplitude, regularity, and sideways amplitude were significantly less responsive (i.e., worse) than in the preferred walking condition. Peak angular velocity, amplitude asymmetry and coordination were not significantly different between these two conditions. In the dual-task walking condition, amplitude asymmetry was significantly more responsive (i.e., better) and main amplitude, peak angular velocity, coordination, sideways amplitude, and cognitive performance were significantly less responsive (i.e., worse) than in the fast walking condition. Regularity was not significantly different between these two conditions.

### Correlations with the medication-induced changes in arm swing

Almost none of the ON-OFF changes in arm swing parameters correlated with any ON-OFF changes of the MDS-UPDRS (part three total score and subscores). The only exceptions were sideways amplitude and MDS-UPDRS rigidity subscore during the preferred speed condition (*P* = 0.018), as well as coordination of arm swing and postural instability and gait disorder score (PIGD) during the dual-task walking condition (*P* = 0.027; Table 3). Several of the ON-OFF changes in the arm swing parameters correlated with the Levodopa equivalent daily dose (LEDD). At preferred speed, main amplitude, peak angular velocity and coordination correlated with LEDD (*P* = 0.005, *P* = 0.004, *P* = 0.015, respectively; Table 3). At fast speed, arm swing asymmetry correlated negatively with LEDD (*P* = 0.001). However, during the dual-tasking condition, none of the ON-OFF changes of the arm swing parameters correlated significantly with the LEDD.

**Table 3:** Correlation coefficients of the changes in arm swing parameters with medication and the changes in MDS-UPDRS III (subscores) with medication, and the LEDD values.

Parameter	MDS-UPDRS III total score			MDS-UPDRS Bradykinesia subscore			MDS-UPDRS Rigidity subscore			MDS-UPDRS Tremor subscore			MDS-UPDRS PIGD subscore			LEDD		
	Pref	Fast	Dual	Pref	Fast	Dual	Pref	Fast	Dual	Pref	Fast	Dual	Pref	Fast	Dual	Pref	Fast	Dual
n	41	41	34	41	41	34	41	41	34	41	41	34	41	41	34	43	43	36
Main amplitude	0.13	−0.16	−0.05	−0.05	0.04	−0.02	−0.07	−0.10	−0.10	0.18	−0.08	−0.19	−0.28	−0.23	−0.19	0.42	0.08	0.19
Peak angular velocity	0.10	−0.17	−0.05	−0.08	0.05	−0.00	−0.08	−0.14	−0.12	0.18	−0.10	−0.20	−0.29	−0.24	−0.18	0.43	0.15	0.16
Asymmetry	0.17	−0.02	0.29	0.22	0.08	0.28	−0.01	−0.03	−0.03	0.06	0.16	0.18	0.14	−0.01	0.26	−0.04	−0.49	−0.18
Coordination	0.05	0.02	−0.26	0.05	0.13	−0.27	−0.17	−0.02	−0.11	0.07	−0.03	−0.09	−0.17	−0.03	−0.39	0.38	−0.11	0.18
Regularity	−0.06	−0.18	−0.22	−0.18	−0.04	−0.26	−0.08	−0.24	−0.13	0.12	−0.01	−0.09	−0.21	−0.04	−0.06	0.13	0.11	−0.03
Sideways amplitude	0.19	0.04	−0.02	0.10	−0.10	−0.03	0.36	0.02	0.22	−0.08	−0.02	0.11	0.15	0.08	0.09	−0.29	0.17	−0.09

*Dual* dual-task walking, *LEDD* Levodopa equivalent daily dose, *MDS-UPDRS* Movement Disorders Society-sponsored revision of the unified Parkinson’s disease rating scale, *PIGD* postural instability and gait disorder, *Pref* preferred speed.

Significant correlations in bold (*P* < 0.05).

## Discussion

This study shows that the effect of dopaminergic medication on arm swing is substantially influenced by the context in which patients with Parkinson’s disease walk. Arm swing during walking improved with dopaminergic medication at preferred walking speed, and it improved even more during fast walking at least for some parameters (main amplitude, peak angular velocity, coordination, and sideways amplitude). However, the responsiveness of dopaminergic medication on arm swing changed drastically by adding a cognitive dual-task to walking compared to preferred and fast walking only, respectively. In the dual-task walking condition, the responsiveness to dopaminergic medication was low for most arm swing parameters, and sideways amplitude got even worse. Only amplitude asymmetry improved, because the amplitude of the more affected arm increased, while the amplitude of the less affected arm decreased, reducing the difference between both arms (Supplementary Fig. 1). A different response to medication in the more and the less affected side was only seen for amplitude and peak angular velocity during the dual-task condition. We suggest that the talking out loud provides rhythmical stimulation that could have a positive effect on the coordination between both arms causing a more symmetrical arm swing pattern. The correlations between the change in arm swing parameters with dopaminergic medication and the LEDD support that the responsiveness to dopaminergic medication is influenced by the context. At preferred walking speed, three-arm swing parameters correlated with LEDD values, at fast speed only one and none during dual-tasking.

The changes in main arm swing amplitude, peak angular velocity, and coordination with medication at preferred speed corresponds with other studies investigating gait aspects in Parkinson’s disease^5,16,30^. In previous studies, looking at gait parameters, it has been seen that mainly the amplitude- and velocity-based measures (step length, gait velocity, step velocity) improved with medication at preferred speed, which is comparable to our results^2,5–7,10^. The reduction in arm swing asymmetry found in this study corresponded with one study^16^, but not with another which was probably due to the inclusion of patients with dyskinesia in that study^5^. Other studies also found effects of medication on gait parameters during more challenging (fast) walking conditions^7,9^. Concerning more complex walking paradigms contradicting results were found^18,19^. One study even found a larger reduction in stride time variability with medication during dual-tasking compared to single tasking^19^. Since gait speed significantly changed between medication states and single- and dual-tasking, these effects could very well be mediated by gait speed. This issue holds also true for studies investigating arm swing. To our knowledge, none of the currently available arm swing studies controlled their results for gait speed, although it is known that arm swing is influenced by this parameter^24,31,32^ and dopaminergic medication increases gait speed^6–8,11^. In this study, many significant Spearman’s correlations of arm swing parameters with gait speed were found, with values reaching up to 0.47 (asymmetry, fast speed) for patients with Parkinson’s disease in OFF medication state and up to 0.53 (main amplitude, dual tasking) for patients with Parkinson’s disease in ON medication state. We therefore recommend to perform this gait speed correction in future studies, otherwise, there may be a risk that gait speed-associated (and not disease state-associated) aspects are measured.

The cognitive performance increased with dopaminergic medication in the single-task condition, but not in the dual-task condition (Fig. 2). This effect was accompanied by significantly more pronounced subtraction task dual-task costs in the medication OFF state compared to the ON state. We interpret these results according to already existing literature^33,34^ in that way that, when patients with Parkinson’s disease perform a dual-task in OFF state, they prioritize the cognitive task. This prioritization of the cognitive task could have detrimental effects on the walking performance.

In the dual-task condition, the cognitive performance, as well as most arm swing parameters did not improve with dopaminergic medication. A possible explanation for this could be the “levodopa overdose hypothesis”^35^. Dopaminergic medication does not target one specific brain area^19^. For example, it affects the mesocorticolimbic pathway, which has a negative effect on cognitive function, including an executive function that is required to control gait in patients with Parkinson’s disease^19,36,37^. This could be a cause for the absent improvement in cognitive performance and most arm swing parameters.

Although clinical assessments are in many aspects different from daily living assessments^38^, studies have shown that more complex clinical assessments correspond relatively well with the average values of daily living assessments^22,39^. Our study shows that the effect of dopaminergic medication on arm swing is rather small or even negative during dual-tasking. This implies that dopaminergic medication might, for this specific and potentially very relevant movement^40^, not be very beneficial in real-life situations. For handwriting, it also has been shown that dopaminergic medication had no effect on the more complex writing tasks, compared to writing down letters or one word repeatedly in patients with Parkinson’s disease^41^. We can thus confirm these findings with another upper limb movement (arm swing), and contribute evidence that the effect of dopaminergic medication should not only be tested under standardized conditions but absolutely must also be tested under daily-relevant situations. It seems possible that these medication effects differ substantially between supervised and daily-life (-relevant) conditions, and to a significant disadvantage for affected patients. The even negative effect of dopaminergic medication on sideways amplitude during dual-tasking could indicate a decrease in dynamic postural stability with dopaminergic medication, which must certainly be investigated in more detail in future studies. Nevertheless, it is possible that the difference in dopaminergic responsiveness due to different walking conditions affects not only the upper but also the lower extremities. During simple static postural stability tasks a positive effect of dopaminergic medication was found^5,42^, when more complex (eyes closed and dual-task conditions) static postural stability tasks were performed there was no effect of dopaminergic medication found^43^. During dynamic postural stability tasks there were also no effects of dopaminergic medication found and in the PIGD subgroup the postural stability even frequently deteriorated with medication^1,44^. Therefore, it seems that dopaminergic medication does not improve the postural stability during complex tasks. Interestingly, this phenomenon could also be seen in other neurotransmitter systems. During preferred speed and simple dual-task walking conditions, patients with Parkinson’s disease, treated with the cholinesterase inhibitor rivastigmine, had a significantly better (reduced) step time variability compared to the placebo-treated group in a simple walking paradigm but there were no significant differences found between the two groups during a complex dual-task walking condition^45^.

None of the ON-OFF changes of arm swing parameters correlated significantly with respective changes of the total MDS-UPDRS III score. This observation strongly argues that arm swing is a movement that is largely independent of “classic” Parkinson’s disease symptoms. This is all the more remarkable as there was no effect observed in any of the three different walking conditions. If this observation can also be confirmed in larger independent studies and cohorts, and this effect is potentially also shown in free-living environments, arm swing parameters in Parkinson’s disease could be used as an easily and frequently detectable complementary sign for disease progression and treatment response in clinical routine and clinical trials. Moreover, there was a positive correlation between the rigidity subscore and sideways amplitude in the preferred walking speed condition (and somewhat less pronounced and not significant in the dual-task walking condition). Rigidity causes the absence or reduction of trunk rotations. Rotations of the thorax are known to contribute to arm swing^46^, therefore with decreased trunk rotations a smaller arm swing amplitude, in both main and sideways direction, was expected. This was, however, not the case for the sideways amplitude. It seems plausible that, due to rigidity, the trunk can contribute less to balance recovery during walking in PD. Consequently, sideways arm swing could serve as a compensatory movement to recover from balance perturbations. To determine whether the sideways amplitude is a parameter for (limitations of) dynamic balance, further research is required. The other significant (negative) correlation observed was between the PIGD items and arm swing coordination during dual-task walking. The postural instability and gait problems could cause a more unstable gait pattern and the arms might be used to compensate for any balance disturbances. Compensatory movements of the arms might negatively influence the timing between the left and right arm. The postural instability can especially be prominent during dual-tasking where patients prioritize the cognitive performance causing a decrease in postural stability according to the “posture second” strategy^33^. This significant negative correlation could speak for the usefulness of this parameter for determining the severity of (and therapy response to) PIGD symptoms, e.g., under everyday conditions^39^.

This study faces limitations. First, participants performed both OFF and ON assessments on the same day and always OFF before ON, therefore fatigue is a possible confounder in this study. However, all study participants were allowed to take breaks at any time during the individual task performance. Second, only patients with mild to moderate disease severity were included, which means that the results cannot be extrapolated to more advanced disease stages.

Taken together, this study shows that the responsiveness of dopaminergic medication on arm swing in people with Parkinson’s disease depends on the context and task complexity. These results should motivate more granular and extensive research in the area of task complexity-influenced responsiveness of mobility aspects to dopaminergic medication in Parkinson’s disease.

## Methods

### Participants

Forty-five patients with a diagnosis of Parkinson’s disease according to the UK Brain Bank Society Criteria^47^ and a Hoehn & Yahr stage between one and three (reflecting mild to moderate disease severity) were recruited at the University Hospital of Tübingen, Germany. Patients with an impaired range of motion of the shoulder due to trauma were excluded as well as patients with dyskinesia, because dyskinesia most probably has a significant and “uncontrollable” influence on gait parameters^5^.

The ethical committee of the Medical Faculty of the University of Tübingen approved this study (715/2011B02). All participants gave a written informed consent prior to testing according to the declaration of Helsinki.

### Data collection

Participants walked a 20 m walkway up and down for 1 min, under three conditions: (i) preferred speed (“Walk at your preferred walking speed”), (ii) fast speed (“Please walk as fast as you can, do not run, do not risk falling”), and (iii) fast speed in combination with a serial subtraction task started from a three-digit number (“Please walk as fast as you can, do not run, do not risk falling, and subtract serial sevens as fast as you can from the number I will shortly tell you”). This serial subtraction task was also separately performed as single-task. All participants performed the assessments first OFF medication (overnight withdrawal from dopaminergic medication) and 30 min to 2 h after medication intake (based on the participant’s feedback when they usually experience best ON) in ON medication condition. In both medication states the motor part of the MDS-UPDRS, part III, was assessed. The MDS-UPDRS part II was also assessed, but only assessed once. The dopaminergic medication the patients took was collected from the medical file to calculate the LEDD^48^. During the assessments, all participants wore an inertial measurement unit with tri-axial accelerometers, gyroscopes, and a magnetometer (128 Hz sample frequency; Opal APDM, Portland, USA) on each wrist and one on the lower back.

### Data processing

All completed straight walking phases of the 1 min walk were extracted (turns were discarded from the data with help of a turn detection algorithm validated for patients with Parkinson’s disease and healthy older adults^49^). The gait speed was calculated by dividing the 20 m walked distance by the time it took to walk those 20 m (based on the turn detection described above). The arm swing parameters from the straight walking phases were extracted with an arm swing algorithm validated for patients with Parkinson’s disease and healthy adults^23^. Arm swing was defined as “a rotational movement of the arm, occurring during walking and running in bipeds with a periodicity of around 1–2 Hz. The hand and arm move freely through space in opposite directions with most of the movement in the sagittal plane of the body frame”^23^. To omit false positives, only arm swings with an amplitude of at least five degrees were taken into account^23^. The first three and last three swings of the straight walking phases were excluded from the analysis so that only steady-state walking phases were considered.

The arm swing algorithm extracts information from both arms, which results in the following parameters (Table 4): main amplitude (amplitude in main swing direction), peak angular velocity, regularity, coordination, and asymmetry^23^. We also included in this analysis sideways amplitude, reflecting the amplitude of the movement during the swing in the direction orthogonal to the main swing direction (movements around the longitudinal axis are not taken into account). Sideways arm swing could be a compensatory movement to get the center of mass back above the base of support. This movement therefore may reflect, as a measure of dynamic postural stability, correction, or adaptation movements during walking^50^. The parameter was calculated from the second component of the principal component analysis^23^. The dual-task costs for the cognitive serial subtraction task were calculated for both medication states^34^.

**Table 4:** Description of the arm swing parameters. Exact calculations of the parameters can be found in^23^.

Parameter	Description
Main amplitude [deg]	The average magnitude of a swing in the main swing direction
Peak angular velocity [deg/s]	The average maximal angular velocity of a swing
Asymmetry [%]	The non-directional difference in main amplitude between both arms (0% left and right arm swing on average with a similar main amplitude; 100% left and right arm swing on average with an entirely different main amplitude)
Coordination (0–1)	A measure for the timing between the left and right arm (1 if both arms move exactly out of phase, e.g. left arm at most forward point and right arm at most backward point; 0 if both arms do not move in a similar rhythm), the calculation is based on a cross-correlation
Regularity angular velocity (0–1)	The similarity of a swing with its neighbouring ipsilateral swings (1 similar; 0 not similar), the calculation is based on an auto-correlation
Sideways amplitude [% of main amplitude]	The average proportion of movement that occurs orthogonal to the main swing direction

### Statistical analysis

Since arm swing is affected by gait speed^24,31,32^, the parameters were corrected for this parameter using a linear regression between gait speed and each arm swing parameter per condition and per (medication) group. All parameters were corrected to their estimated value at 1 m/s. Wilcoxon signed-rank tests were used to analyse the effects of dopaminergic medication on the arm swing parameters. Tests were two-tailed with a significance level of 0.05.

To analyse the effect of medication on the cognitive performance during single-tasking and dual-tasking Wilcoxon signed-rank tests were performed. As well as for the effect of medication on the dual-task costs.

The standardized response mean (SRM) was calculated by dividing the average of the change ($\bar x_{{{{{{\mathrm{change}}}}}}}$) by the standard deviation of the change in a certain parameter:

1 $$\bar x_{change} = \frac{1}{N}\mathop {\sum}\limits_{i = 1}^N {\left( {x_{i,{{{{{\mathrm{on}}}}}}} - x_{i,{{{{{\mathrm{off}}}}}}}} \right)}$$

2 $$SRM = \frac{{\bar x_{change}}}{{\sqrt {\frac{1}{{N - 1}}\mathop {\sum}\nolimits_{i = 1}^N {\left| {\left( {x_{i,{{{{{\mathrm{on}}}}}}} - x_{i,{{{{{\mathrm{off}}}}}}}} \right) - \bar x_{{{{{{\mathrm{change}}}}}}}} \right|^2} } }}$$

*N* represents the amount of participants and *x*_*i*_ the arm swing parameter of each participant in ON or OFF state, with 0.20 ≤ *SRM* < 0.50 representing a small, 0.50 ≤ *SRM* < 0.80 a moderate and *SRM* ≥ 0.80 a large responsiveness to dopaminergic medication^5^.

The significances of dopaminergic medication effects between the three walking conditions were analysed with a repeated measures ANOVA. A Greenhouse-Geisser correction was performed when the assumption of sphericity was violated. *P* < 0.05 was considered significant. Post hoc testing was performed with Bonferroni corrections to control for type 1 errors.

Spearman correlations were performed to test associations between ON-OFF effects of arm swing parameters and clinical scores (total MDS-UPDRS III, and MDS-UPDRS subscores: bradykinesia (items 3.4, 3.5, 3.6, 3.8^51^), rigidity (item 3.3), tremor (items 2.10, 3.15, 3.16, 3.17, 3.18^52^), and PIGD (items 2.12, 2.13, 3.10, 3.11, 3.12^53^), and LEDD. Significance of these exploratory analyses was considered when *P* < 0.05.

### Reporting Summary

Further information on research design is available in the Nature Research Reporting Summary linked to this article.

## Supplementary information

Supplementary Information

Reporting Summary

## Data Availability

The data from this study are available upon reasonable request.

## Code availability

The algorithm to extract the arm swing parameters can be accessed and downloaded online (https://github.com/EWarmerdam/ArmSwingAlgorithm)^23^. The algorithm was developed with Matlab 2017a.

## References

[1] Bloem BR (1996). Influence of dopaminergic medication on automatic postural responses and balance impairment in Parkinson’s disease. Mov. Disord..

[2] Rosqvist K (2018). Levodopa effect and motor function in late stage Parkinson’s disease. J. Parkinsons. Dis..

[3] Hong M, Earhart GM (2010). Effects of medication on turning deficits in individuals with Parkinson’s Disease. J. Neurol. Phys. Ther..

[4] Zach H, Dirkx M, Pasman JW, Bloem BR, Helmich RC (2017). The patient’s perspective: the effect of levodopa on Parkinson symptoms. Park. Relat. Disord..

[5] Curtze C, Nutt JG, Carlson-Kuhta P, Mancini M, Horak FB (2015). Levodopa is a double-edged sword for balance and gait in people with Parkinson’s disease. Mov. Disord..

[6] Marxreiter F (2018). Sensor-based gait analysis of individualized improvement during apomorphine titration in Parkinson’s disease. J. Neurol..

[7] McNeely ME, Duncan RP, Earhart GM (2012). Medication improves balance and complex gait performance in Parkinson disease. Gait Posture.

[8] Rochester L, Baker K, Nieuwboer A, Burn D (2011). Targeting dopa-sensitive and dopa-resistant gait dysfunction in Parkinson’s disease: Selective responses to internal and external cues. Mov. Disord..

[9] Bryant MS (2011). Gait variability in Parkinson’s disease: influence of walking speed and dopaminergic treatment. Physiol. Behav..

[10] Bryant MS, Rintala DH, Hou JG, Lai EC, Protas EJ (2011). Effects of levodopa on forward and backward gait patterns in persons with Parkinson’s disease. NeuroRehabilitation.

[11] Blin O, Ferrandez AM, Pailhous J, Serratrice G (1991). Dopa-sensitive and Dopa-resistant gait parameters in Parkinson’s disease. J. Neurol. Sci..

[12] Ferrarin M, Rizzone M, Lopiano L, Recalcati M, Pedotti A (2004). Effects of subthalamic nucleus stimulation and L-dopa in trunk kinematics of patients with Parkinson’s disease. Gait Posture.

[13] Hoskovcova M (2015). Predicting falls in Parkinson disease: What is the value of instrumented testing in off medication state?. PLoS One.

[14] Bowes S (1990). Determinants of gait in the elderly parkinsonian on maintenance levodopa/carbidopa therapy. Br. J. Clin. Pharmacol..

[15] Bryant MS, Rintala DH, Hou J, Collins RL, Protas EJ (2016). Gait Variability in Parkinson’s Disease: Levodopa and Walking Direction. Acta Neurol. Scand..

[16] Sterling NW (2015). Dopaminergic modulation of arm swing during gait among Parkinson’s disease patients. J. Parkinsons. Dis..

[17] Hamacher D, Herold F, Wiegel P, Hamacher D, Schega L (2015). Brain activity during walking: A systematic review. Neurosci. Biobehav. Rev..

[18] Elshehabi, M. et al. Limited effect of dopaminergic medication on straight walking and turning in early-to-moderate parkinson’s disease during single and dual tasking. *Front. Aging Neurosci*. **8**, 4(2016). 10.3389/fnagi.2016.00004 PMC4728201 26858638

[19] Dagan M (2021). Dopaminergic therapy and prefrontal activation during walking in individuals with Parkinson’s disease: does the levodopa overdose hypothesis extend to gait?. J. Neurol..

[20] Hackney ME, Earhart GM (2017). The Effects of a secondary task on forward and backward walking in Parkinson disease. Physiol. Behav..

[21] Alcock L, Galna B, Perkins R, Lord S, Rochester L (2018). Step length determines minimum toe clearance in older adults and people with Parkinson’s disease. J. Biomech..

[22] Atrsaei, A. et al. Gait speed in clinical and daily living assessments in Parkinson’s disease patients: performance versus capacity. *npj Park. Dis*. **7**, 24 (2021). 10.1038/s41531-021-00171-0 PMC7935857 33674597

[23] Warmerdam, E. et al. Quantification of arm swing during walking in healthy adults and Parkinson’s disease: Wearable sensor-based algorithm development and validation. *Sensors.* **20**, 5963 (2020). 10.3390/s20205963 PMC7590046 33096899

[24] Mirelman, A. et al. Effects of aging on arm swing during gait: the role of gait speed and dual tasking. *PLoS One* **10**, e0136043 (2015). 10.1371/journal.pone.0136043 PMC4549059 26305896

[25] Baron EI, Miller Koop M, Streicher MC, Rosenfeldt AB, Alberts JL (2018). Altered kinematics of arm swing in Parkinson’s disease patients indicates declines in gait under dual-task conditions. Park. Relat. Disord..

[26] Plate A (2015). Normative data for arm swing asymmetry: How (a)symmetrical are we?. Gait Posture.

[27] Huang X (2012). Both coordination and symmetry of arm swing are reduced in Parkinson’s disease. Gait Posture.

[28] Lewek MD, Poole R, Johnson J, Halawa O, Huang X (2010). Arm swing magnitude and asymmetry during gait in the early stages of Parkinson’s disease. Gait Posture.

[29] Roggendorf J (2012). Arm swing asymmetry in Parkinson’s disease measured with ultrasound based motion analysis during treadmill gait. Gait Posture.

[30] Crenna P (2008). Influence of basal ganglia on upper limb locomotor synergies. Evidence from deep brain stimulation and L-DOPA treatment in Parkinson’s disease. Brain.

[31] Romkes J, Bracht-Schweizer K (2017). The effects of walking speed on upper body kinematics during gait in healthy subjects. Gait Posture.

[32] Fang, X. & Jiang, Z. Three-dimensional thoracic and pelvic kinematics and arm swing maximum velocity in older adults using inertial sensor system. *PeerJ*. **8**, e9329 (2020). 10.7717/peerj.9329 PMC7350916 32704440

[33] Bloem BR, Grimbergen YAM, van Dijk JG, Munneke M (2006). The ‘posture second’ strategy: A review of wrong priorities in Parkinson’s disease. J. Neurol. Sci..

[34] Hobert, M. A. et al. Poor trail making test performance is directly associated with altered dual task prioritization in the elderly - baseline results from the trend study. *PLoS One* **6**, e27831 (2011). 10.1371/journal.pone.0027831 PMC3218043 22114705

[35] Vaillancourt DE, Schonfeld D, Kwak Y, Bohnen NI, Seidler R (2013). Dopamine overdose hypothesis: evidence and clinical implications. Mov. Disord..

[36] Lord S, Rochester L, Hetherington V, Allcock LM, Burn D (2010). Executive dysfunction and attention contribute to gait interference in ‘off’ state Parkinson’s Disease. Gait Posture.

[37] Plotnik M, Dagan Y, Gurevich T, Giladi N, Hausdorff JM (2011). Effects of cognitive function on gait and dual tasking abilities in patients with Parkinson’s disease suffering from motor response fluctuations. Exp. Brain Res..

[38] Warmerdam E (2020). Long-term unsupervised mobility assessment in movement disorders. Lancet Neurol..

[39] Hillel, I. et al. Is every-day walking in older adults more analogous to dual-task walking or to usual walking? Elucidating the gap between gait performance in the lab and during 24/7 monitoring. *Eur. Rev. Aging Phys. Act*. **16**, 6 (2019). 10.1186/s11556-019-0214-5 PMC6498572 31073340

[40] Meyns P, Bruijn SM, Duysens J (2013). The how and why of arm swing during human walking. Gait Posture.

[41] Zham P (2019). Effect of levodopa on handwriting tasks of different complexity in Parkinson’s disease: a kinematic study. J. Neurol..

[42] Beuter A, Hernández R, Rigal R, Modolo J, Blanchet PJ (2008). Postural sway and effect of levodopa in early Parkinson’s disease. Can. J. Neurol. Sci..

[43] Workman CD, Thrasher TA (2019). The influence of dopaminergic medication on balance automaticity in Parkinson’s disease. Gait Posture.

[44] Pelicioni PHS (2018). Head and trunk stability during gait before and after levodopa intake in Parkinson’s disease subtypes. Exp. Gerontol..

[45] Henderson EJ (2016). Rivastigmine for gait stability in patients with Parkinson’s disease (ReSPonD): a randomised, double-blind, placebo-controlled, phase 2 trial. Lancet Neurol..

[46] Pontzer H, Holloway JH, Raichlen DA, Lieberman DE (2009). Control and function of arm swing in human walking and running. J. Exp. Biol..

[47] Gibb WRG, Lees AJ (1988). The relevance of the Lewy body to the pathogenesis of idiopathic Parkinson’s disease. J. Neurol. Neurosurg. Psychiatry.

[48] Tomlinson CL (2010). Systematic review of levodopa dose equivalency reporting in Parkinson’s disease. Mov. Disord..

[49] Pham, M. H. et al. Algorithm for turning detection and analysis validated under home-like conditions in patients with parkinson’s disease and older adults using A 6 degree-of-freedom inertial measurement unit at the lower *Back. Front. Neurol*. **8**, 135 (2017). 10.3389/fneur.2017.00135 PMC5385627 28443059

[50] Curtze C, Hof AL, Postema K, Otten B (2011). Over rough and smooth: amputee gait on an irregular surface. Gait Posture.

[51] Stebbins GT, Goetz CG (1998). Factor structure of the Unified Parkinson’s Disease Rating Scale: motor Examination section. Mov. Disord..

[52] Forjaz MJ (2015). Proposing a Parkinson’s disease–specific tremor scale from the MDS-UPDRS. Mov. Disord..

[53] Stebbins GT (2013). How to identify tremor dominant and postural instability/gait difficulty groups with the movement disorder society unified Parkinson’s disease rating scale: comparison with the unified Parkinson’s disease rating scale. Mov. Disord..

