# Supplementary material for: Arm swing responsiveness to dopaminergic medication in Parkinson’s disease depends on task complexity
Source: NPJ Parkinsons Dis. 2021 Oct 5;7:89. doi: 10.1038/s41531-021-00235-1 (PMC8492858; doi:10.1038/s41531-021-00235-1)
Supplement: Supplementary file 1 — Supplementary Information [file 41531_2021_235_MOESM1_ESM.pdf]

Supplementary material

Supplementary Table 1. Results from the Wilcoxon signed rank test to analyse the effect of dopaminergic medication on arm swing as well as the difference with controls

	**Preferred**						**Fast**						**Dual-task**					
	OFF-ON		OFF-Controls		ON-Controls		OFF-ON		OFF-Controls		ON-Controls		OFF-ON		OFF-Controls		ON-Controls	
**n**	43		43		43		43		43		43		36		36		36	
	Z	*P*	*Z*	*P*	*Z*	*P*	*Z*	*P*	*Z*	*P*	*Z*	*P*	*Z*	*P*	*Z*	*P*	*Z*	*P*
**Main amplitude**	-2.44	**0.015**	-0.83	0.405	1.04	0.299	-5.69	**<0.001**	-2.96	**0.003**	2.86	**0.004**	0.56	0.649	-1.38	0.167	-1.57	0.116
**Peak angular velocity**	-2.15	**0.032**	-0.95	0.340	0.74	0.461	-5.71	**<0.001**	-2.64	**0.008**	2.06	**0.039**	0.14	0.888	-1.07	0.285	-1.13	0.258
**Asymmetry**	3.72	**<0.001**	2.61	**0.009**	0.39	0.697	-2.78	**0.005**	-4.26	**<0.001**	-3.12	**0.002**	2.24	**0.025**	2.78	**0.005**	0.20	0.845
**Coordination**	-1.41	0.158	0.31	0.313	0.52	0.600	-5.45	**<0.001**	-2.00	**0.045**	3.87	**<0.001**	-1.01	0.313	-2.65	**0.008**	-2.72	**0.007**
**Regularity**	-3.47	**<0.001**	-4.41	**<0.001**	-3.67	**<0.001**	-0.06	0.952	-3.85	**<0.001**	-3.44	**<0.001**	1.87	0.062	0.83	0.405	-0.22	0.826
**Sideways amplitude**	2.50	**0.012**	0.81	0.419	-0.60	0.546	5.49	**<0.001**	5.52	**<0.001**	-2.54	**0.011**	-2.86	**0.004**	-1.38	0.167	0.97	0.330

Supplementary Table 2. The results of the repeated measures ANOVA that compared the dopaminergic medication responsiveness per walking condition

	**Repeated Measures ANOVA**			**Post hoc testing**					
				pref-fast		pref- dual		fast-dual	
	df	*F*	*P*	*t*	*P*	*t*	*P*	*t*	*P*
**Main amplitude**	2	42.72	**<0.001**	-6.36	**<0.001**	2.63	**0.032**	8.99	**<0.001**
**Peak angular velocity**	2	24.51	**<0.001**	-4.74	**<0.001**	2.09	0.120	6.83	**<0.001**
**Asymmetry**	1.62	12.78	**<0.001**	-3.52	**0.003**	1.38	0.521	4.90	**<0.001**
**Coordination**	1.58	11.61	**<0.001**	-4.06	**<0.001**	0.21	1.000	4.27	**<0.001**
**Regularity**	1.64	4.23	**0.026**	1.85	0.206	2.87	**0.017**	1.02	0.937
**Sideways amplitude**	1.55	33.99	**<0.001**	3.73	**0.001**	-4.50	**<0.001**	-8.23	**<0.001**

In bold the significant results. Post hoc testing was performed with Bonferroni correction. df = degrees of freedom; pref = preferred.

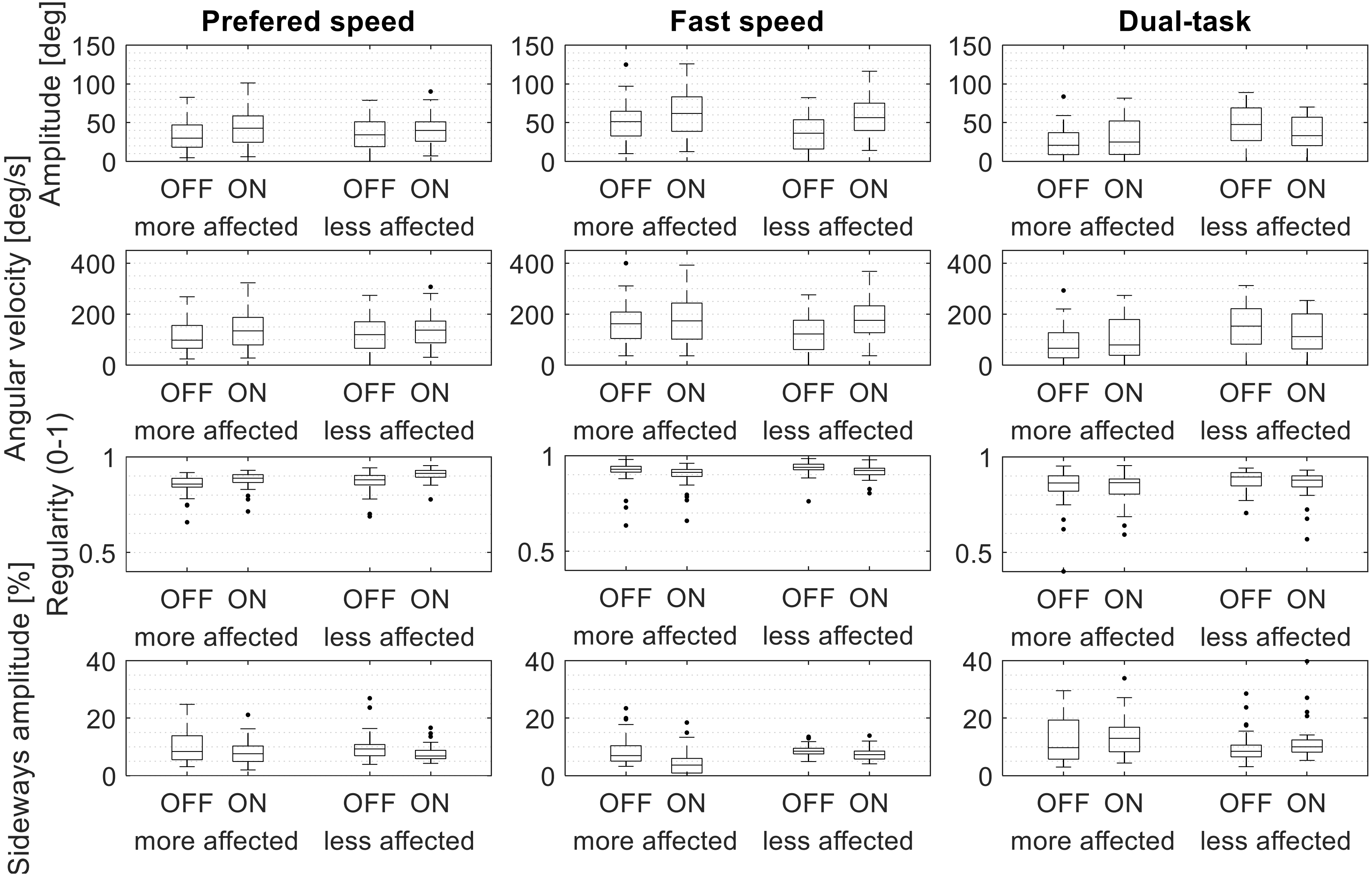

Supplementary Figure 1. The arm swing parameters for the more and less affected side during the different medication states and different walking conditions.
