# Supplementary material for: Arm swing responsiveness to dopaminergic medication in Parkinson’s disease depends on task complexity
Source: NPJ Parkinsons Dis. 2021 Oct 5;7:89. doi: 10.1038/s41531-021-00235-1 (PMC8492858; doi:10.1038/s41531-021-00235-1)
Supplement: Supplementary file 2 — Reporting Summary [file 41531_2021_235_MOESM2_ESM.pdf]

nature research

Corresponding author(s): Elke Warmerdam
NPJPARKD-00725

Last updated by author(s): Jul 19, 2021

Reporting Summary

Nature Research wishes to improve the reproducibility of the work that we publish. This form provides structure for consistency and transparency in reporting. For further information on Nature Research policies, see our Editorial Policies and the Editorial Policy Checklist.

Statistics

For all statistical analyses, confirm that the following items are present in the figure legend, table legend, main text, or Methods section.

n/a	Confirmed	
☐	☒	The exact sample size (n) for each experimental group/condition, given as a discrete number and unit of measurement
☐	☒	A statement on whether measurements were taken from distinct samples or whether the same sample was measured repeatedly
☐	☒	The statistical test(s) used AND whether they are one- or two-sided<br>*Only common tests should be described solely by name; describe more complex techniques in the Methods section.*
☒	☐	A description of all covariates tested
☐	☒	A description of any assumptions or corrections, such as tests of normality and adjustment for multiple comparisons
☐	☒	A full description of the statistical parameters including central tendency (e.g. means) or other basic estimates (e.g. regression coefficient) AND variation (e.g. standard deviation) or associated estimates of uncertainty (e.g. confidence intervals)
☐	☒	For null hypothesis testing, the test statistic (e.g. F, t, r) with confidence intervals, effect sizes, degrees of freedom and P value noted<br>*Give P values as exact values whenever suitable.*
☒	☐	For Bayesian analysis, information on the choice of priors and Markov chain Monte Carlo settings
☒	☐	For hierarchical and complex designs, identification of the appropriate level for tests and full reporting of outcomes
☒	☐	Estimates of effect sizes (e.g. Cohen's d, Pearson's r), indicating how they were calculated

Our web collection on statistics for biologists contains articles on many of the points above.

Software and code

Policy information about availability of computer code

Data collection	no software was used
Data analysis	The algorithm to extract the arm swing parameters is freely available online (https://github.com/EWarmerdam/ArmSwingAlgorithm).

For manuscripts utilizing custom algorithms or software that are central to the research but not yet described in published literature, software must be made available to editors and reviewers. We strongly encourage code deposition in a community repository (e.g. GitHub). See the Nature Research guidelines for submitting code & software for further information.

Data

Policy information about availability of data

All manuscripts must include a data availability statement. This statement should provide the following information, where applicable:

- Accession codes, unique identifiers, or web links for publicly available datasets
- A list of figures that have associated raw data
- A description of any restrictions on data availability

The data from this study are available upon reasonable request.

Field-specific reporting

Please select the one below that is the best fit for your research. If you are not sure, read the appropriate sections before making your selection.

☒ Life sciences ☐ Behavioural & social sciences ☐ Ecological, evolutionary & environmental sciences

For a reference copy of the document with all sections, see nature.com/documents/nr-reporting-summary-flat.pdf

Life sciences study design

All studies must disclose on these points even when the disclosure is negative.

Sample size	45 Parkinson patients were included. This group size should be sufficient to explore the effect of medication on arm swing during different tasks, since per task at least 6000 arm swings from Parkinson patients were analysed.
Data exclusions	All participants that performed a walking assessment in both ON and OFF medication state were included. For certain statistical analysis the participants that did not perform all three walking assessments in both medication states were excluded.
Replication	The arm swing algorithm that was used for the data analysis was validated against a gold standard reference system, therefore replication of the results should lead to the same conclusions.
Randomization	Randomization was not relevant for this study. The patients were all in one group that performed the assessment twice (OFF and ON medication).
Blinding	Blinding was not possible since the Parkinson patients were always measured OFF medication (overnight withdrawal of medication) before they were measured ON medication.

Reporting for specific materials, systems and methods

We require information from authors about some types of materials, experimental systems and methods used in many studies. Here, indicate whether each material, system or method listed is relevant to your study. If you are not sure if a list item applies to your research, read the appropriate section before selecting a response.

Materials & experimental systems

n/a	Involved in the study	
☒	☐	Antibodies
☒	☐	Eukaryotic cell lines
☒	☐	Palaeontology and archaeology
☒	☐	Animals and other organisms
☐	☒	Human research participants
☐	☒	Clinical data
☒	☐	Dual use research of concern

Methods

n/a	Involved in the study	
☒	☐	ChIP-seq
☒	☐	Flow cytometry
☒	☐	MRI-based neuroimaging

Human research participants

Policy information about studies involving human research participants

Population characteristics	Participants needed to have a diagnosis of Parkinson's disease according to the UK Brain Bank Society Criteria and a Hoehn & Yahr stage between 1 and 3. Both man and woman were included.
Recruitment	Patients with a diagnosis of Parkinson’s disease were recruited at the University Hospital of Tübingen, Germany.
Ethics oversight	The ethical committee of the Medical Faculty of the University of Tübingen

Note that full information on the approval of the study protocol must also be provided in the manuscript.

Clinical data

Policy information about clinical studies

All manuscripts should comply with the ICMJE guidelines for publication of clinical research and a completed CONSORT checklist must be included with all submissions.

Clinical trial registration	DRKS00022058
Study protocol	https://www.trend-studie.de/english/study-protocol/

Data collection	The data was collected at the University Hospital of Tübingen, Germany. The Parkinson patients were always measured OFF medication in the morning and about one hour later ON medication.
Outcomes	The primary outcome measures were predefined as the effect of medication on arm swing.
